# Developmental Foreign Accent Syndrome: Report of a New Case

**DOI:** 10.3389/fnhum.2016.00065

**Published:** 2016-03-10

**Authors:** Stefanie Keulen, Peter Mariën, Peggy Wackenier, Roel Jonkers, Roelien Bastiaanse, Jo Verhoeven

**Affiliations:** ^1^Clinical and Experimental Neurolinguistics, Vrije Universiteit BrusselBrussels, Belgium; ^2^Center for Language and Cognition Groningen, Rijksuniversiteit GroningenGroningen, Netherlands; ^3^Department of Neurology and Memory Clinic, ZNA Middelheim General HospitalAntwerp, Belgium; ^4^Department of Language and Communication Science, City University LondonLondon, UK; ^5^Computational Linguistics and Psycholinguistics Research Center, Universiteit AntwerpenAntwerp, Belgium

**Keywords:** Developmental Foreign Accent Syndrome, FAS, Developmental apraxia of speech, speech disorder, constructional dyspraxia, SPECT

## Abstract

This paper presents the case of a 17-year-old right-handed Belgian boy with developmental FAS and comorbid developmental apraxia of speech (DAS). Extensive neuropsychological and neurolinguistic investigations demonstrated a normal IQ but impaired planning (visuo-constructional dyspraxia). A Tc-99m-ECD SPECT revealed a significant hypoperfusion in the prefrontal and medial frontal regions, as well as in the lateral temporal regions. Hypoperfusion in the right cerebellum almost reached significance. It is hypothesized that these clinical findings support the view that FAS and DAS are related phenomena following impairment of the cerebro-cerebellar network.

## Introduction

Foreign accent syndrome (FAS) is a relatively rare motor speech disorder in which segmental and prosodic speech alterations cause patients to be perceived as non-native speakers of their mother tongue (Blumstein et al., 1987; Ingram et al., 1992; Lippert-Gruener et al., 2005; Pyun et al., 2013; Tran and Mills, 2013). In some cases, there is a reversion to a previously acquired language variety (Seliger et al., 1992; Kwon and Kim, 2006). In 2010, Verhoeven and Mariën provided a taxonomical classification of this speech disorder and defined three main types of FAS: a neurogenic, psychogenic and mixed type (Verhoeven and Mariën, 2010a). Neurogenic FAS is further subdivided into an acquired and a developmental^1^ variant. The current article focuses on developmental FAS, which is one of the rarest etiological subtypes of FAS. To the best of our knowledge only two case studies have been published between 1907 and 2014 (Mariën et al., 2009). The first case was a 29-year-old female native speaker of Belgian Dutch who was diagnosed with FAS and developmental apraxia of speech (DAS). The second patient was a 7-year-old boy, who presented with FAS in the context of specific language impairment (SLI) of the phonological-syntactic type (Mariën et al., 2009).

Although the number of documented developmental FAS cases has remained low, accent change has been (anecdotally) reported in relation to neurodevelopmental disorders, especially autism of the Asperger-type (Attwood, 1998; Ghazziudin, 2005; Tantam, 2012). However, in these reports, the neurobiological relationship between the speech characteristics and the developmental disorder was not addressed in detail. Hence, it is possible that FAS is much more common in a population with developmental disorders than current statistics indicate. This article presents a new case of developmental FAS in combination with DAS: a neurologically based speech disorder that affects the planning/programming of phonemes and articulatory sequences as language develops, in the absence of any neuromuscular impairment (Crary, 1984; McNeill and Kent, 1990; Smith et al., 1994). The patient is a 17-year-old right-handed native speaker of Belgian Dutch (Verhoeven, 2005) who presented with articulatory problems and an accent, which was perceived as French or “Mediterranean” by family, medical staff and acquaintances. A neurological and neuropsychological assessment was carried out and both an MRI and a SPECT were performed. Furthermore, the patient's speech was analyzed phonetically. Since this occurrence of FAS is linked to a programming disorder, the hypothesis of FAS as a possible subtype of apraxia of speech will be addressed in detail.

## Background

The assessment presented in this article was carried out following the principles of the standard clinical neurolinguistic work-up of patients with speech- and/or language disorders at ZNA Middelheim hospital in Antwerp (Belgium). The patient's parents provided written informed consent to report the patient's medical data.

A 17-year-old, right-handed, native speaker of Belgian Dutch consulted the department of Clinical Neurolinguistics of ZNA Middelheim Hospital because of persisting articulation difficulties resulting in accented speech. The patient indicated that listeners identified him as a non-native speaker of Dutch with a French or “Mediterranean” accent. He was born at term after normal gestation and labor, and there had been no perinatal or postnatal problems. Medical history was unremarkable. According to “WHO child growth standards” acquisition of gross motor milestones was normal. He could sit without support at 5.5 months (mean = 6.0; SD = 1.1), stand with assistance at 7 months (mean = 7.6; SD = 1.4) and walk independently at the age of 11 months (mean = 12; SD = 1.8 months). He was able to independently ride a bicycle without support at the age of 4.0 years. By the age of 4–5 years he had developed a clear right-hand preference.

Except for a deviant development of articulation skills, developmental milestones were normal, including non-motor speech and language ability. The patient did not present with any pervasive developmental disorder and no family history of developmental disorders or learning disabilities was reported. There were no clinical indications for a psychiatric disorder. The parents and close relatives stated that the patient was in perfect mental health. The patient was not under any medication at the time of examination. Speech therapy was started at the age of 5 years and discontinued at the age of 10 because of a lack of therapeutic progress. The parents were monolingual speakers of Dutch. The patient had successfully finished primary school and obtained above average results in the 3rd grade of secondary school. Neurological investigations, including EEG recordings, were normal. MRI of the brain revealed no lesions at the supra- and infratentorial level. There was no brain atrophy.

**Figure 1:**
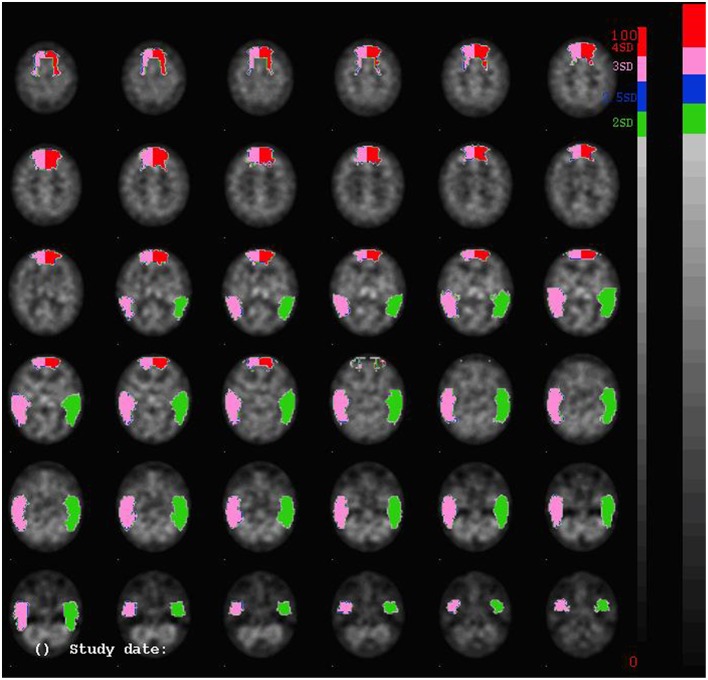
**SPECT-findings demonstrating a significant decrease of perfusion bilaterally in the prefrontal and medial frontal regions, as well as in the lateral temporal regions**.

A quantified Tc-99m-ECD SPECT study was carried out. 740 MBq (20 mCi) Tc-99m-ECD was administered to the patient by means of a previously fixed butterfly needle while he was sitting in a quiet dim room, eyes open and ears unplugged. Acquisition was started 40 min after injection using a three-headed rotating gamma camera system (Triad 88; Trionix Research Laboratory, Twinsburg, Ohio, USA) equipped with lead super-fine fanbeam collimators with a system resolution of 7.3 mm FWHM (rotating radius 13 cm). Projection data were accumulated in a 128 x 64 matrix, pixel size 3.56 mm, 15 s per angle, 120 angles for each detector (3° steps, 360° rotation). Projection images were rebinned to parallel data, smoothed and reconstructed in a 64 × 64 matrix, using a Butterworth filter with a high cut frequency of 0.7 cycles/cm and a roll-off of 5. No attenuation or scatter correction was performed. Trans-axial images with a pixel size of 3.56 mm were anatomically standardized using SPM and compared to a standard normal and SD image obtained from ECD perfusion studies in a group of 15 normally educated healthy adults consisting of 8 men and 7 women with an age ranging from 45 to 70 years. This normal image was created by co-registration of each normal study to the SPECT template image of SPM using the “normalize” function in SPM. At the same time, the global brain uptake of each study was normalized. On the mean image, 31 ROI's were drawn and a 31 ROI template was created. Using the normalized studies and the 31 ROI template, the mean normal uptake and SD value (= 1 Z-score) in each ROI was defined. Patient data were normalized using SPM in the same way and the perfusion uptake in each ROI was calculated. From this uptake, the mean uptake and SD value of the normal database, the Z-score for each region can be calculated. A regional Z-score of >2.0 is considered significant. SPECT findings are illustrated in Figure 1.

A significant bilateral hypoperfusion distributed in the medial prefrontal regions (right: −3.48 SD; left: −4.97 SD) and in both lateral temporal regions (right: −3.17 SD; left: −2.17 SD) was found. Decreased perfusion in the left inferior medial frontal region (−1.65 SD), the right inferior lateral frontal region (−1.62) and the right cerebellar hemisphere (−1.52) nearly reached significance.

### Neuropsychological investigations

In-depth neuropsychological assessment consisted of a range of formal tests including the Wechsler Adult Intelligence Scale, 4th Ed., Dutch version (WAIS-IV-NL) (WAIS-IV: Wechsler, 2008; WAIS-IV-NL: Kooij and Dek, 2012), the Bourdon-Vos Test (Vos, 1998), the Wisconsin Card Sorting Test (WCST) (Heaton et al., 1993), the Stroop Color-Word Test (Stroop, 1935; Golden, 1978), the Trail Making Test (TMT) (Reitan, 1958), the Rey-Osterrieth figure (Rey, 1941; Osterrieth, 1944), the praxis subtests of the Hierarchic Dementia Scale (HDS) (Cole and Dastoor, 1987), the Beery Developmental Test of Visual-Motor Integration, 5th Ed. (Beery and Beery, 2004) and the Test of Visual-Perceptual Skills, third edition (TVPS-3) (Martin, 2006). Neurolinguistic assessment consisted of the Boston Naming Test (Kaplan et al., 1983; Belgian norms (Dutch): Mariën et al., 1998), the Clinical Evaluation of Language Fundamentals (Dutch version) (Semel et al., 2003) and the Dudal Spelling Tests (Dudal, 1998, 2004). Test results are summarized in Table 1.

**Table 1:** **Overview of the neuropsychological test results**.

**Test**	**Scaled score (raw score)**	**Percentile**	**Mean**	**SD**	***Z-score***
**INTELLIGENCE (WAIS IV)**					
Wechsler Full Scale IQ (FSIQ)	119		100	15	+1.27
Wechsler Verbal Comprehension Scale	122		100	15	+1.47
Similarities	13		10	3	+1
Vocabulary	15		10	3	+1.67
Information	14		10	3	+1.33
Wechsler Perceptual Reasoning Scale	112		100	15	+0.8
Block Design	10		10	3	0
Matrix Reasoning	14		10	3	+1.33
Visual Puzzles	12		10	3	+0.67
Wechsler Working Memory Scale	117		100	15	+1.33
Digit Span	12		10	3	+0.67
Arithmetic	14		10	3	+1.33
Wechsler Processing Speed Scale	103		100	15	+0.2
Symbol Search	11		10	3	+0.33
Coding	10		10	3	0
**MEMORY**					
WMS-R Visual Memory Index	120		100	15	+1.33
Figure Memory	(8/10)				
Visual Paired Associates I	(18/18)				
Visual Reproduction I	(39/41)	92			
WMS-R Verbal Memory Index	126		100	15	+1.73
Logical Memory I	(42/50)	98			
Verbal Paired Associates I	(22/24)				
WMS-R General Memory Index	131		100	15	+2.06
WMS-R Delayed Recall Index	>138		100	15	>+2.53
Logical Memory II	(40/50)	97			
Visual Paired Associates II	(6/6)				
Verbal Paired Associates II	(8/8)				
Visual Reproduction II	(39/41)	95			
**ATTENTION**					
Bourdon-Vos Test Speed	(9.87′′)	50	50		0
Accuracy	(2)	75	1.40	0.89	0.67
**EXECUTIVE FUNCTIONS**					
Wisconsin Card Sorting Test					
Nr of categories realized	(1)				
Nr of trials	(128)				
Stroop Color-Word Test					
Card I	(45′′)	50	45		0
Card II	(55′′)	50	55		0
Card III	(96′′)	30	95.70	0.58	−0.52
Trail Making Test					
Part A	(21′′)	>90			
Part B	(43′′)	>90			
**LANGUAGE**					
Boston Naming Test	(55/60)		47.89	4.31	+1.65
EMT-B	9		10	3	−0.33
EMT-B item 50	9		10	3	−0.33
Dudal spelling					
Words	(31/40)	55			+0.13
Sentences	(33/40)	80			+0.84
Total	(64/80)	70			+0.52
CELF-IV-NL					
Recalling Sentences	11	63	10	3	+0.33
Formulated Sentences	14	91	10	3	+1.33
Word Definitions	13	84	10	3	+1
Word Classes Receptive	16	98	10	3	+2
Word Classes Expressive	13	84	10	3	+1
Word Classes Total	15	95	10	3	+1.67
Understanding Spoken Paragraphs	14	91	10	3	+1.33
Sentence Assembly	14	91	10	3	+1.33
Semantic Relationships	13	84	10	3	+1
Core Language Index	121		100	15	+1.4
Receptive Language Index	129		100	15	+1.93
Expressive Language Index	118		100	15	+1.2
Language Content Index	125		100	15	+1.67
Language Structure Index	122		100	15	+1.47
**PRAXIS**					
Rey Complex Figure	(28/36)		35	3	−2.33
HDS Ideatonal: It. 5	(10/10)		9.79	0.17	+1.24
HDS Ideomotor: It. 3	(10/10)		9.94	0.23	+0.26
**VISUAL COGNITION**					
Beery Visual-Motor Integration	78		100	15	−1.47
Beery Visual Perception	94		100	15	−0.4
Beery Motor Coordination	73		100	15	−1.8

General cognitive skills as measured by the WAIS-IV showed a high average full scale IQ level (FSIQ = 119) and average to above average results for each of the subscales. Problems primarily concerned abstract concept formation: shifting and maintaining goal-oriented cognitive strategies in response to changing environmental contingencies was abnormal as the patient only succeeded to complete 1 category within 128 trials (WCST). The planning and construction of a complex geometrical form (Rey-Osterrieth Figure) was abnormal. On the Beery Developmental Test of Visual-Motor Integration the patient obtained borderline results for visual-motor integration skills (−1.4 SD) and for visual-motor coordination (−1.8 SD). Visual perception was normal. Articulation and prosody in conversational and spontaneous speech were clearly abnormal. The patient produced several substitution errors as well as omissions and additions during spontaneous conversation. Oral-verbal diadochokinesis was within normal limits, whereas rapid repetition of polysyllabic words was hesitant. Visual confrontation naming (BNT) and semantic verbal fluency were normal as well. Indices on CELF-IV-NL (Semel et al., 2003) were all above average. No grammatical errors, and lexical retrieval difficulties were observed. Spelling of words and sentences (Dudal spelling) was normal. The isolated motor speech impairments consisted of substitution errors for consonants (affecting place and manner of articulation: e.g., “groepje*n*” instead of “groepje*s*”: little groups, the use of a uvular trill instead of an alveolar trill) and vowels (affecting vowel distinctiveness), difficulties initiating words (“ra. ra. ra… geraak”: get somewhere) and omissions of consonants (“geraa” instead of “geraak,” “pagia” instead of “pagina”: page). These errors are consistent with a diagnosis of DAS (see also “*phonetic analysis”* below).

### Phonetic analysis

A perceptual error analysis of a 1:36 min spontaneous speech sample consisting of 397 words was carried out. This was supplemented by an acoustic analysis of some key aspects of speech. As far as consonant production is concerned, occasional voicing errors were observed (*stravde* for *strafte:* past tense of “punished”). It was furthermore striking that the speaker used a uvular trill instead of the alveolar trill: although both are acceptable realizations of the trill in Dutch, the alveolar trill is the more common variant in the Brabantine geographical region of origin of this speaker. It is precisely the usage of a uvular trill that is typical of French non-native speakers of Dutch.

**Figure 2:**
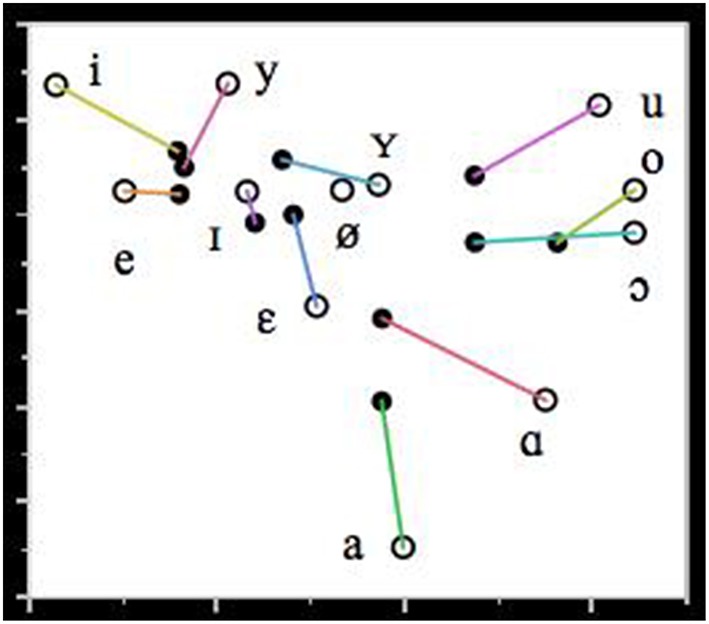
**Mean formant values of F1 and F2 (in Hz) of the Dutch vowels in the FAS speaker (filled circles) and the control group (unfilled circles)**. The lines connect the vowel realizations of the FAS speaker and the control group.

With respect to vowel articulation, various distortions were observed. In order to quantify these deviations, the formant frequencies of the 358 peripheral vowels in the speech sample were measured by means of the signal processing software PRAAT (Boersma and Weenink, 2015). The instances of schwa were not analyzed. The mean formant values of the FAS vowels are illustrated in Figure 2. They have been correlated to the vowel formants of a group of 5 male native control speakers of Dutch from the same geographical region as the FAS speaker. The formant values of the control speakers were obtained in a data collection independent of this investigation, which is described in more detail in Adank et al. (2004).

Figure 2 shows that with respect to vowel production: (1) there is a significant degree of vowel reduction and (2) a substantial erosion of vowel distinctiveness particularly in the front vowels. The observed vowel reduction, i.e. the more central realization of the vowels with respect to the control vowels, can be accounted for by the fact that the vowels in the FAS speaker and the control group have been recorded in different communicative settings. The vowels of the control group were recorded in a structured reading task in which the vowels were positioned in a prominent utterance position in order to attract sentence stress. This leads to a more careful pronunciation of the vowels and gives rise to more peripheral formant values than in spontaneous speech. Hence, the vowel reduction observed in the FAS speaker is unlikely to be contributory to the impression of a foreign accent.

The erosion of the distinctiveness of some vowels in the FAS speaker is particularly noticeable in the close front region of the vowel space: there is very little qualitative difference between /i/, /y/ and /e/, and between /i/, /ε/ and /y/. This smaller distinctiveness cannot be explained by the regional accent of the speaker (Verhoeven and Van Bael, 2002): therefore, it is not unreasonable to assume that this lack of distinctiveness may have contributed to the perception of a foreign accent.

At the suprasegmental level, several dimensions were studied. First, speech rate was investigated from two perspectives, that is as speech rate and articulation rate. Speech rate is expressed as the number of syllables per second, including silent and filled pauses, while articulation rate is quantified as the number of syllables per second including filled pauses, but excluding silent pauses (Verhoeven et al., 2004). In this FAS speaker, speech rate was 3.83 syll/s and articulation rate amounted to 4.79 syll/s. This compares well to a control group of unimpaired native speakers of Dutch who had a speaking rate and articulation rate of 3.89 syll/s and 4.23 syll/s respectively (Verhoeven et al., 2004). From this, it can be concluded that this speaker's speech is generally very fluent and it is precisely the dissociation in fluency between FAS and AoS that has previously been mentioned as one of the hallmark features distinguishing both speech disorders from each other (Aaronson, 1990; Moen, 2000).

The next dimension that was investigated was the speaker's speech rhythm, which was quantified by means of the pairwise variability index (PVI) proposed by Low et al. (2000). This index is based on measures of vowel durations (vocalic PVI) and the duration of the intervocalic intervals (intervocalic PVI). In this speaker, the vocalic PVI amounted to 48: this is considerably lower than 65.5, which is the reference value for Dutch suggested in Grabe and Low (2002). However, it is very close to 43.5, which is the reference value for French. This suggests that the speaker's rhythm is more French-like (syllable-timed) than Dutch (stress-timed) and this may have contributed to the impression of a French accent.

Finally, the speaker's intonation was investigated along the same lines as Verhoeven and Mariën (2010a). As far as the mean pitch and the excursion sizes of the pitch movements in the contours are concerned, it was found that the speaker's mean pitch is 110.5 Hz while his pitch range amounts to 5.85 semi-tones. This agrees rather well with averages for male native speakers of Dutch suggested in ‘t Hart et al. (1990). The internal composition of the pitch contours was analyzed by means of the stylization method proposed by ‘t Hart et al. (1990). This method uses speech analysis and synthesis techniques to replace the original F0 contours by means of a minimal combination of straight lines which are perceptually equivalent. This method eliminates microprosodic variation and provides an insight in the internal structure of pitch contours. For more information about the application of this method to the analysis of speech pathology the interested reader is referred to Verhoeven and Mariën (2010b).

Application of the stylization method revealed 4 different pitch contours. The first one consists of a prominence-lending rising pitch movement (symbolized as 1) immediately followed by a prominence-lending fall (symbolized as A) in the same syllable. This (1-A) pattern occurred 49 times (36.6 %) in the patient's speech sample and it was always correctly associated with the most prominent syllable in the utterance. The second contour is one in which the rising and falling pitch movements 1 and A are aligned with two different prominent syllables: the two movements are connected by means of a stretch of high pitch. The occurrence of this contour is confined to the last two prominent syllables in sentences. This contour was used 13 times (9.7%) by the speaker: all instances were well-formed and agreed with the distributional restrictions of this contour. The third contour is another variant of 1-A in which the first sentence accent is realized by means of a prominence-lending rising pitch movement (1) and the last accent is marked by means of a prominence-lending falling pitch movement (A). Any intervening accents are marked by means of a half fall (symbolized as E) and this gives rise to a typical terrace contour. The speaker used this contour 8 times (6%). The fourth contour is a continuation contour in which the accent is realized by means of a prominence-lending rising pitch movement. The pitch remains high and is then reset to a lower level in order to mark a syntactic boundary (symbolized as B). This is the standard continuation contour, which indicates that the utterance is not finished yet. This contour was used 64 times (47.8%). The 1-B contour did not always coincide with syntactic boundaries, but it was noticed that often individual words within a larger syntactic unit were realized with this contour.

The frequencies of the contours in this speech sample were compared to reference frequencies for spontaneous Dutch reported in Blaauw (1995), who carried out a perceptual analysis of instruction dialogs in 5 speakers. This comparison revealed that the frequency of occurrence of all the speaker's contours was very similar to the reference values suggested in Blaauw (1995), except for the 1B contour, which was significantly more frequent than in unimpaired speech. A similar observation was reported in Verhoeven and Mariën (2010a) and Kuschmann (2010) for neurogenic acquired FAS.

## Discussion

### Semiological resemblances between FAS and DAS

This patient presented with isolated developmental motor speech problems consistent with a diagnosis of FAS and DAS. Previous research has shown that FAS may result from a compensation strategy by patients showing apraxia-like features in speech production (Whiteside and Varley, 1998). It is argued that the same can be assumed for DAS patients. Fluency has been mentioned as one of the key characteristics distinguishing AoS (Van der Merwe, 2009; Duffy, 2013) and FAS patients, and it seems that this semiological distinction also holds for DAS patients. Furthermore, DAS (and AoS) is often characterized by attainment of phonological sequences, whereas FAS is characterized by deviations of individual speech sounds (Moen, 2000).

This patient demonstrated many of the key features associated with DAS (Shriberg et al., 1997a,b; Nijland et al., 2003; Peter and Stoel-Gammon, 2005; McCauley and Strand, 2008; Morgan and Vogel, 2009; Terband et al., 2009; see also: *neuropsychological investigations*). Some of these errors are typical segmental errors which have also been observed in other FAS cases. However, this patient did not show the typical “trial-and-error” behavior which is regularly noted in DAS patients (Stackhouse, 1992; Moen, 2000, 2006; Ozanne, 2005; Hall et al., 2007; Terband et al., 2011). The analysis of suprasegmental features for this case provided supplementary evidence against the idea that FAS is primarily a prosodic deficit: the only remarkable feature was a syllable-timed speech rhythm and the excessive use of the 1B (continuation) contour. Speech and articulation rate, mean pitch (parameter of intonation) and the general shape of the intonation contours were normal.

### Planning deficits: Crossing speech boundaries

The hypothesis of FAS as a subtype of AoS, has previously been described in a physiological (Moen, 2000) and a cognitive perspective (Whiteside and Varley, 1998). This patient was also investigated from both perspectives. Cognitive assessment demonstrated (selective) executive disturbances (deviant scores on the Wisconsin Card Sorting Test and low results on the Stroop Task-card III) and distorted planning and organization in the visuo-spatial domain. However, the patient obtained average to above-average results on other executive tasks (such as the digit span and TMT-B, for instance). Comparison with the cognitive profile of the previously published cases of developmental FAS revealed a comparable discrepancy. The neuropsychological test results of the first patient published by Mariën et al. (2009) demonstrated a low average performance IQ as well as depressed scores for digit span and TMT-A and B. Scores for the WCST and Stroop task on the other hand, were well within the normal range. In their second patient, only severe syntactic deficits affecting language processing were retained. All other cognitive test results were in the average range or above. The results were consistent with a diagnosis of SLI of the phonological-syntactic type. Both the results of this patient and the first patient described by Mariën et al. (2009) go against the finding that WCST scores are a predictor for TMT-B performance, claiming that both tests give expression to attentional set-shifting problems (Sánchez-Cubillo et al., 2009). Some studies have claimed that correlations between the Stroop interference and TMT-B constitute evidence of a shared expression of inhibitory control (Chaytor et al., 2006). Other studies have contradicted such a correlation. For instance, Sánchez-Cubillo et al. (2009) analyzed 41 Spanish-speaking healthy participants and found that TMT-A scores primarily tap visuo-perceptual abilities and visual search (a significant amount of the variance in multiple regression analysis was predicted by the WAIS-III Digit Symbol score), whereas the TMT-B was primarily informed by working memory and only then by task-switching ability (their correlation with the Stroop Interference Task was nulled in the multiple regression analysis).

Functional neuroimaging with SPECT in this patient revealed a decreased perfusion in the anatomo-clinically suspected brain regions involving the bilateral prefrontal cortex, the medial frontal regions and the cerebellum. On the basis of lesion studies, research has linked damage affecting the prefrontal cortex (PFC) to impaired executive functioning (Robinson et al., 1980; Yuan and Raz, 2014). Yuan and Raz (2014) carried out a literature survey about the anatomo-functional correlates of executive functions and showed that increased PFC volume in healthy subjects correlated (positively) with scores on the WCST. Buchsbaum et al. (2005) also found that perfusion in the bilateral PFC significantly increases during performance of tasks requiring executive planning and control. However, the value of the WCST as an exclusive indicator of frontal dysfunction remains a matter of debate. Chase-Carmichael et al. (1999) for instance, have contested the value of the WCST as an indicator of frontal pathology in a pediatric population (age 8–18). For their study, they classified children according to the affected brain area(s) (left hemisphere, right hemisphere, or bilateral frontal, extrafrontal, or multifocal/diffuse regions of brain dysfunction) regardless of the etiology (stroke, brain trauma, tumor, seizures, neurofibromatosis, lupus, myelomeningocele, and cognitive changes of unknown origin). Results did not support the assumption that WCST performance is more impaired in frontal lesions than extrafrontal or multifocal/diffuse lesions. However, they classified all patients with frontal lobe dysfunction together and did not take into consideration differences in the affected *sub*-regions. Moreover, they argue that dysfunction in certain sub-regions (e.g., medial frontal regions) of the frontal lobe in the left hemisphere leads to lower performance on the WCST (Drewe, 1974; Grafman et al., 1986). Still, their study confirmed that patients with left-hemisphere damage generally perform weaker than patients with right hemisphere damage. For adult stroke patients, the same conclusion holds (Jodzio and Biechowska, 2010).

This patient also obtained borderline scores on the motor integration and coordination subtests of the Beery-Buktenica Developmental Test of Visual-Motor Integration; which is a test administered to evaluate the integration of visual perception and co-ordination of fine motor skills in drawing (Beery, 1989). The patient also obtained a low score on the reproduction of the Rey Complex Figure (28/36). It was concluded from these results that the patient had spatial planning, visual structuring and copying (drawing) problems. The patient was diagnosed with a constructional dyspraxia following execution and planning problems of frontal origin.

Because the scores obtained for Block Design, Visual Puzzles (visuo-constructional tests), and the visual perception subtest of Beery-Buktenica Test (perceptual skills) were in the average, unimpaired range, it is hypothesized that the main deficit occurs in the programming phase of the relevant motor movements prior to execution of grapho-motor tasks (Del Giudice et al., 2000). According to the model proposed by Grossi and Angelini (Grossi, 1991, see also: Grossi and Trojano, 1998) the copying of drawings requires (1) a *visuo-spatial analysis* of the geometrical and spatial aspects of the figure to be copied, as well as a scan of the repertoire of internalized figures drawn in the past, (2) the *formulation of a drawing plan*, stored in the working memory (visuo-spatial sketchpad, Baddeley and Hitch, 1974) containing the integration of visuo-spatial representations into the required motor actions (programming phase) (3) the *execution of the grapho-motor movements* (4) and finally the *control of these movements* (see also: Denes and Pizzamiglio, 1998). Since this patient obtained a maximum score on the retention of visual material during neuropsychological testing, it is plausible that the impairment is situated *after* the instauration of the figure in the visuo-spatial sketchpad (working memory). This model is developed along the same lines as the speech sensorimotor control models (Van der Merwe, 2009). In short, the problem might be situated in the second phase of planning and programming. Furthermore, this patient did not demonstrate a hypoperfusion in the (superior) parietal region, where graphomotor plans are stored. Yet a significant hypoperfusion was found in the area circumscribing the (bilateral) prefrontal cortex, the area where graphomotor plans are programmed/integrated for execution (Mariën et al., 2013). Disorders of skilled movements, as well as underdeveloped constructional abilities have been noted in the context of DAS (Yoss and Darley, 1974; McLaughlin and Kriegsmann, 1980; Maassen, 2002).

### The hypothesis of a cortico-cerebellar network dysfunction

The frontal executive dysfunctions in conjunction with the SPECT findings lead to the hypothesis that the pattern of hypoperfusions reflects significant involvement of the cerebro-cerebellar functional connectivity network (Meister et al., 2003; Mariën et al., 2006, 2013; Mariën and Verhoeven, 2007; Moreno-Torres et al., 2013). Cerebellar involvement in speech disorders, including FAS and AoS, has previously been proposed from the viewpoint of the cerebellum as a coordinator of speech timing (see also: De Smet et al., 2007). Also, the phonetic analysis of our patient's speech gave evidence for semiological resemblances between DAS and FAS. However, one of the most striking differences between both conditions, namely the fluency aspect was equally confirmed for our patient. These findings provide support for the hypothesis that FAS may be a mild subtype of AoS as well the developmental cognate (Whiteside and Varley, 1998; Moen, 2000, 2006; Fridriksson et al., 2005; Mariën et al., 2006, 2009; Kanjee et al., 2010).

In hindsight, diffusion tensor imaging (DTI) might be of added value to identify structural changes to the white matter tracts which make up and connect with the cortico-cerebellar tract. DTI voxel-based morphometry was unfortunately not carried out in this patient. However, it could help to further clarify the pathophysiological substrate of neurodevelopmental disorders and should be considered in future research on developmental FAS.

## Concluding remarks

A new case of developmental FAS with DAS and a visuo-spatial planning disorder was presented. From a semiological as well as structural and physiological point of view, the hypothesis of a connection between FAS and DAS seems plausible in this case. Moreover, the conjunction between the speech impairment and frontal executive deficits, supported by SPECT findings provide further evidence for a potentially primary role of the cerebro-cerebellar network in both disorders. However, one of the main characteristics of DAS is trial-and-error behavior. This was not attested since the patient could adequately self-correct whenever production errors were made. Therefore, the hypothesis is put forward that FAS is a *mild* subtype of AoS, even when both are developmental in nature.

## Author contributions

All authors listed, have made substantial, direct and intellectual contribution to the work, and approved it for publication.

### Conflict of interest statement

The authors declare that the research was conducted in the absence of any commercial or financial relationships that could be construed as a potential conflict of interest.

## Footnotes

^1^As the focus of the current article is the developmental subtype of foreign accent syndrome, the interested reader is referred to Verhoeven and Mariën (2010a) for a comprehensive discussion of the FAS taxonomy.
